# COVID-19 and its implications for obstetrics and gynecology practice in Africa

**DOI:** 10.11604/pamj.2021.38.15.25520

**Published:** 2021-01-07

**Authors:** Timothy Abiola Olusesan Oluwasola, Oluwasomidoyin Olukemi Bello

**Affiliations:** 1 Department of Obstetrics and Gynecology, College of Medicine, University of Ibadan/University College Hospital, Ibadan, Nigeria

**Keywords:** COVID-19, implications, obstetrics, gynecology, practice, Africa

## Abstract

Having to cope with corona virus disease 2019 (COVID-19) is likely to create imbalances in health care provision in the obstetrics and gynecology practices in Africa where most countries still battle with high rate of maternal morbidities and mortalities as well as poor or inadequate quality gynecological care. COVID-19 has spread to the continents of the world including all African nations since it was first reported in Wuhan, China in December 2019. Its impact and implications on the obstetrics and gynecology practice in Africa are yet to be fully explored. Routine essential services are being disrupted; therefore, giving rise to the need to redeploy the already limited health personnel across health services in Africa. This is an attempt to discuss the potential implications for obstetrics and gynecologic practice in Africa.

## Introduction

Corona virus disease 2019 (COVID-19) is a novel public health problem threatening the life of nearly 20 million people globally and is caused by a new human corona virus (SARS-CoV-2) previously known as 2019-nCov [1,2]. In Africa, COVID-19 infection was first reported on 14^th^ February, 2020 in Egypt and community transmission was suspected by the following month [3]. The main route of transmission of the virus is through droplet spread and via contact from contaminated surfaces to mucosal surfaces [4-6]. The virus may also become aerosolized during certain airway interventions and cardiopulmonary resuscitation [6]. The risks of exposure for healthcare providers increase when medical emergencies occur. Meanwhile, of all emergencies, obstetrical emergencies in patients who are suspected or confirmed COVID-19 cases are particularly complex. However, a systematic approach to management of these emergencies would contribute significantly to lowering the risk of healthcare providers´ exposure to the virus [7].

## Methods

The articles used were searched from PubMed, Google Scholar, Scopus databases using terms such as: COVID-19/SARS-CoV-2 in Africa, impact of corona virus on the African health sector and COVID-19 in obstetrics and gynecology care in Africa. Also, reference lists of published articles including reviews and documents on databases such as WHO, UNFPA and FIGO were browsed for potential articles.

## Current status of knowledge

**Burden of COVID-19:** currently, about 15-20 per cent of COVID-19 cases have been found among women in the reproductive age group [8]. Therefore, adverse consequences from outbreaks of COVID-19 with involvement of sexual and reproductive health of vulnerable women and young people in Africa should be anticipated considering the absence of effective therapy, vaccine or pre-existing immunity [9]. However, more than two-thirds of the world´s maternal deaths occur in sub-Saharan Africa with inadequate access to quality care during pregnancy, delivery or postpartum period being the identified leading cause [10]. Despite commitment to improving maternal health through quality care, the uptake of maternal health services in sub-Saharan Africa remains low [10]. The level of unmet need for protection and hygiene products for female frontline health workers who make up the majority of health workers involved in COVID-19 care and treatment in Africa is unknown [11].

The spate of nosocomial COVID-19 infection and exhaustion of the health workforce is disheartening [12]. For surgeons, obstetricians and anesthesiologists, the major challenges included ensuring provision of emergency and essential surgery and obstetric care while preserving precious resources, minimizing exposure of health care workers and preventing onward transmission [13]. There are scarce resources for treating critically ill patients with COVID-19 in Africa and there are fewer than 2000 ventilators spread across the 41 African countries that reported to the World Health Organization in April, 2020. With only five in each of Democratic Republic of Congo and Liberia, Somalia´s health ministry has no ventilator, while Central African Republic has three and South Sudan has four. Nigeria, with a high population, has less than 100 ventilators [14]. Recently, the total number of confirmed cases of COVID-19 in Nigeria hit over 13000 as at 9^th^ of June 2020 despite an abysmally low rate of testing and more than tripled over a two months period with 46,577 confirmed cases and 945 deaths by 9^th^ August 2020 [15].

**Effect of COVID-19 on health workers and facilities:** clinic appointments are infrequent in low-income settings because of limited availability of healthcare providers and patients tend to wait long hours at crowded clinic waiting areas for antenatal care, contraceptive counseling or other reproductive health services. This portends great danger for the patients, their providers and the society as there is a higher risk of infection transmission [16]. Additionally, in most low and middle income countries (LMICs), acute and emergency maternal and reproductive health services have the potential to be hit the hardest in view of the limited facilities for isolation areas for the assessment and care for women in labour including their newborn. Lifesaving procedures, from caesarean deliveries and emergency obstetric care to abortion care are likely to be delayed due to lack/shortage of personal protective equipment (PPEs), staff redeployment and other potential infrastructural inadequacy. Furthermore, women who have to spend time recovering in hospital wards in the LMICs are habitually reliant on relatives for food and care, thus making isolation and infection control measures difficult and thereby increasing the potential risks of spreading the disease [16].

The safety of the medical front liners in which women make up an about 70% of the world´s global health and social sector workforce is currently being compromised through contact with high-risk environments and lack of PPEs [17]. In a recent study conducted in Uganda, it was reported that the health care workers in Makerere University Teaching Hospitals have sufficient knowledge on the transmission, diagnosis and prevention of the transmission of COVID-19 [18]. However, in spite of the knowledge of this disease, some midwives in some African countries (Ghana, Gambia and Malawi) have stated that they are faced with fear and new challenges as the COVID-19 disease spreads in the Africa continent [19]. Adequate knowledge of COVID-19 among care givers will avert negative attitudes and promote positive preventive and therapeutic practices. In addition, modification of routine clinics to include staggered appointments, longer appointment dates and telemedicine where available have become necessary options to pursue in consideration of the current challenges coupled with possible deployment of staff to places where acute care are needed.

### Implication of the pandemic on selected obstetrics and gynecologic practices

**Family planning services:** in Africa, family planning programs have remained fragile, majorly requiring supportive political leadership, especially in Western and Central African countries [20,21]. In several urban regions, the number of women of reproductive age is still large and increasing. About 214 million women of reproductive age in Africa were estimated to have an unmet need for modern contraception in 2017, which accounts for 84 per cent of all unintended pregnancies [22]. In a recent statement by the International Federation of Gynecology and Obstetrics (FIGO), the urgent need to expand postpartum family planning services, promote self-care family planning methods, lift barriers to accessing contraception and improve dissemination of information to access contraception by implementing telemedicine were identified. Furthermore, the need to anticipate and address likely supply chain needs and challenges was specified [23]. The FIGO statement on family planning services released during the COVID-19 pandemic will, to a reasonable extent, help curb unmet needs for family planning services in developing countries and Africa at large [23]. With many families staying longer at home because of the lockdown in several countries, the need for contraceptive and family planning advice is apparently essential. If family planning services are made more available and accessible irrespective of the pandemic, there will be improvement in maternal and child health. Furthermore, sexually transmitted infections will be prevented and there will be a reduction in the incidence of unintended pregnancies and unsafe abortion.

**Antenatal care, deliveries and newborn care:** the pandemic and the response to the pandemic are affecting both the provision and utilization of reproductive, maternal, newborn and child health services. Demand for these services is theoretically likely to decline in hospitals equipped for such services as concerns over COVID-19 transmission alter the perceived benefit-risk calculations for individuals deciding to seek care [24]. Unfortunately, there is tendency to have a surge in the number of women patronizing the traditional birth attendants (TBAs) and hence it is necessary to anticipate the potential challenges associated with such care. There will be need to explore the possible reasons why pregnant women may stay away from health facilities during this COVID-19 pandemic so as to be able to provide scientific solutions that will help to reduce the resultant effects of such staying away on the maternofetal health.

Several pregnant women who need antenatal care are unsure whether to attend a clinic during this pandemic and this situation is similar to the experiences of some African countries such as Liberia, Guinea and Sierra Leone during the 2014-2016 Ebola epidemics [17]. A rise in maternal mortality rate was recorded during and after the outbreak as women stayed away from medical facilities due to quarantine restrictions and misconceptions about the virus transmission such that many of them subsequently had unsupervised home deliveries. New mothers also failed to attend postnatal appointments, with attendance levels remaining low even after the epidemic had passed [17]. This reduction in antenatal clinic attendance and delivery with a skilled birth attendant could further increase the present high rate of maternal and fetal morbidities and mortalities in sub-Saharan Africa. In addition, identifying COVID-19 positive mothers have a significant implication for rooming postpartum mothers with or near one another and in ensuring safe transfer between hospital units. It is important to ensure that mothers, especially those who are positive for COVID-19 (COVID+) but asymptomatic are accurately identified and triaged thereby preventing nosocomial transmission of the infection among the postpartum women. Generally, the use of universal SARS-CoV-2 testing in all pregnant patients presenting for delivery is quite essential and should be encouraged although the risk of transmission during the asymptomatic stage should be noted. The potential advantages of universal testing include the ability to use COVID-19 status to determine hospital isolation practices and bed assignments as well as taking informed decision on neonatal care and guiding the use of PPEs. Access to such clinical data will provide an important opportunity to protect mothers, babies and health care teams during these challenging times [25]. Unfortunately, this appears to be a mirage in most health facilities in Africa where the available test kits are out rightly inadequate for the population at a higher risk of COVID-19 infection.

In 2002-2003, the severe acute respiratory syndrome (SARS) emerged while the Middle East Respiratory Syndrome (MERS), was first reported in 2012. These previous outbreaks had significant impact on the sexual and reproductive health and rights of people at different levels vis-à-vis individual, systems and societal levels. MERS and SARS are known to cause adverse pregnancy outcomes such as abortion, prematurity, fetal growth restriction and maternal death [26]. Since the emergence of the current pandemic, pregnant women and senior citizens are the populations which are most susceptible as well as being more likely to have complications and even progress to severe illness. Although, there have been reported cases of COVID-19 infection in neonates, vertical transmission from mother to child is yet to be confirmed despite theoretical possibilities [27-31]. It was shown that when infected women breastfed while donning mask, their babies had very low risk of contracting the disease but breastfeeding without nose mask exposed some babies to the disease at one day and three-day postpartum period [30]. Although a small-sample-size study that recently tested breast milk found no positive tests for SARS-CoV-2, suggesting that the virus does not pass into breast milk [32], Tam *et al*. and Groß *et al*. in published case reports detected the presence of SARS-CoV-2 RNA in breast milk [33,34]. In a reported case from China, one pregnant woman required mechanical ventilation and a caesarean delivery at 30 weeks gestation [35] while fetal distress and preterm births were reported in some other cases where infection occurred in the third trimester [36]. Experience of COVID-19 in pregnancy is currently limited in Africa; however, several recommendations for the management of pregnant women with or without the disease have been proposed [37].

**Endoscopic procedures:** endoscopic procedures potentially put all care givers involved at risk of inhalation and conjunctival exposure from bio aerosol [38]. In addition, there have been issues pertaining to increased risks of transmission of COVID-19 during gynecological laparoscopic surgery but this is yet to be ascertained. However, the universal testing may not be practical in all settings but screening and testing should be employed as per local protocols [38]. Risk of infection from performing surgery in either way does not differ in terms of open surgery or laparoscopy. Nonetheless, it should be noted that minimally invasive approaches shorten the recovery and hospitalization period of the patients. Protective equipment and recommendations about any type of surgery that may have a risk of aerosolization should be taken into consideration [39,40]. For the African region where laparoscopic surgeries are still evolving and robust evidence of increased risk of viral transmission is lacking [38], it is imperative for the laparoscopic surgeons to consider restricting their number of cases and adopting medical treatment as much as possible while deferring non-emergency cases. Open laparotomy has been recommended in order to reduce the risk of exposure of the healthcare providers. There is need to have a common goal in combating COVID-19 most especially when practicing in a low resource setting with numerous challenges such as constraints of getting respirator masks and PPE, state of the art theatre environment/operating rooms to prevent this disease and scarcity in getting a patient tested to confirm her status before necessary surgery to avoid increased transmission of the virus through aerosols [41].

**Gynecology oncology practice:** cancers and multiple benign conditions have been considered as high-risk in the COVID-19 emergency by the centers for disease control and prevention. Higher risk of death is likely in people living with cancer sequel to the unintended consequences of changes in health service provision as well as due to physical and/or psychological effects of social distancing and economic upheaval [42]. During this pandemic, only urgent and emergency medical services are guaranteed to the extent that the typical cancer treatment timing is no longer definite. Measures of cancer screening as well as follow up care are going to be negatively affected. Moreover, most patients in the LMICs have innate challenges with cancer care which are likely to be worsened by the measures taken to prevent or limit spread of the virus [43]. In a brief communication, Mandato and Aguzzoli reported that deciding which ovarian cancer patient to submit to surgery and which to delay owing to the impossibility of guaranteeing a postsurgical intensive care bed is a main dilemma faced by gynecological oncologists [44].

There is the possibility for increased mortality among people with cancer and multiple morbidities due to the COVID-19 emergency thus demonstrating dramatic changes in cancer services. In fact, the real-life treatment is gradually moving away from best practice and this serves as a potential for worldwide crises in cancer management should the pandemic persist. Basic guidelines for management of gynecological cancer patients are presented in Table 1 and this is likely to work with the African population excellently well [45]. Using innovative interventions to reduce inpatient stay in hospital has been pivotal in managing this crisis powered by the rapid uptake of telemedicine technology. Such interventions are likely to gain momentum worldwide giving rise to a new healthcare system where women can undergo diagnostic testing and be followed up by their caring clinicians at distance. However, their use needs to be rationalized since not all aspects of antenatal and postnatal care can be delivered using telemedicine [46]. Furthermore, decreasing one-on-one or face-to-face antenatal appointments might increase pregnancy complications in high-risk groups such as victims of domestic violence, those with mental health issues and other deprived populations with limited or no access to advanced technology [47]. Also, part of the challenge is the limited number of health facilities in Africa that can consult through institutional telemedicine. Developing institutional telemedicine is a potential gain from this outbreak and should be explored.

**Table 1:** alternative strategies for managing gynecological cancers/malignancies during the COVID-19 pandemic

Malignancies	Management
Uterine corpus	Instances where surgery is not possible, oral progesterone and use of the levonorgestrol- secreting intrauterine system are alternative options; similarly, primary radiotherapy is an effective therapy where available
Ovarian and fallopian tubes	Surgery may be deferred in low risk women while in advanced widespread disease, neoadjuvant chemotherapy, with surgery after 3 cycles of treatment followed by another 3 cycles is considered appropriate
Cervical cancers	In early-stage cervical cancer, surgery remains the primary intervention but if surgery is delayed, then radiotherapy with or without concomitant chemotherapy could be the best therapeutic option
Vulvar cancers	Resection of the tumours is successful in easing pain associated with vulvar cancer; sometimes it may be possible to undertake surgery under local anesthetic; if need be, groin lymphadenectomy should be deferred until a time that is safer for the patient

Abortion is regarded as an essential component of comprehensive health care due to its time-sensitive nature. The American College of Obstetricians and Gynecologists (ACOG) and other gynecology societies have issued joint statements, supporting access to abortion services during the COVID-19 pandemic [48]. In order to decrease risk of exposure and transmission, alternative strategies such as timely referral and remote prescription are important options to be adopted. Meanwhile, if there are no risk factors for ectopic pregnancy and patient has regular menstrual cycle with a known last menstrual period, then assessment, counselling and consent can be done by video or telephone. Mifepristone and misoprostol can also be self-administered at home in the interim [48].

**Elective gynecologic surgeries:** elective surgeries are usually done in non-urgent conditions in order to improve the quality of life (QoL) of patients who have indications for surgical management and optimize their clinical outcomes [49]. ACOG in conjunction with other gynecology societies have also issued a joint statement on the suspension of elective surgeries during the COVID-19 pandemic. Meanwhile, if the delay will impact patient health and cause harm, then the surgery should be performed as scheduled [48]. While following stipulated guidelines on COVID-19 infection and surgical intervention, suspending elective and non-urgent surgical procedures based on the health resources of each country and on the response capacity of each health system in particular is an important benchmark for African countries [50]. According to a recent report by FIGO on restarting elective surgery, considerations should be based on local epidemiological evidence. For instance, elective surgery can restart in a given location if the expected peak of SARS-CoV-2 infection will not occur in the 14-day period following an elective procedure or if the rate of new cases has been steadily decreasing for the preceding 14 days [50].

**Sexually transmitted infections:** sexually transmitted infections (STIs) are caused by bacteria, viruses or parasites that are transmitted via unprotected sex (vaginal, anal or oral) and skin to skin genital contact. Based on current evidence, COVID-19 infection is transmitted via contact with droplets from the nose and mouth, including the saliva of an infected person, which can happen through close contact with others. This means there is a significant risk of transmitting the virus through kissing and physical touching if one person has the virus. There is also evidence that the virus is present in stool such that licking around the anal areas may also be a way for easy transmission [51]. It is unknown if immunosuppression by HIV infection will further worsen an individual´s risk for COVID-19 thereby implying that until more is known, additional precautions for all people with advanced HIV or poorly controlled HIV, should be employed. All people living with HIV should take the necessary precautions to protect themselves from corona virus and ensure that they are adhering properly to their antiretroviral treatment [52]. Women´s choices and rights to sexual and reproductive healthcare should be respected irrespective of COVID-19 status. All countries must strike a fine balance between protecting health, minimizing economic and social disruption, and respecting human rights [53].

**Assisted reproductive techniques:** the potential effects of COVID-19 infection on assisted reproductive treatments demand attention. Considering the initial data and lack of comprehensive knowledge on the pathogenesis of SARS-CoV-2 during pregnancy, human reproduction societies have recommended postponing the embryo transfers and withholding initiation of new treatment cycles. The American Society for Reproductive Medicine (ASRM) recommended that during the corona virus pandemic there should be suspension of initiation of new treatment cycles, including ovulation induction, intrauterine inseminations, invitro fertilization and non-urgent gamete cryopreservation. Secondly, cancellation of all embryo transfers whether fresh or frozen should be considered. Thirdly, there should be continued care for patients who are currently “in-cycle” or who require urgent stimulation and cryo-preservation. Lastly, elective surgeries and non-urgent diagnostic procedures should be suspended, in-person interactions should be minimized while utilization of telehealth should increase [54].

New evidence must be considered carefully in order to apply these recommendations and to guide assisted reproductive treatments [55]. The International Federation for Fertility Societies (IFFS) recommended that patients who are considering pregnancy or who are currently undergoing fertility therapies should consult with their personal physician for planning further steps [56]. As concluded by Monteleone *et al*. deciding between initiating, resuming or postponing assisted reproductive treatments depends more strongly on the need to abide with social isolation and other regulations/interventions necessary for reduction of risk of exposure to the virus rather than on the COVID-19 infection as well as its potential effects during pregnancy, bearing in mind potential emotional effects on the patients [55].

## Conclusion

**Figure 1:**
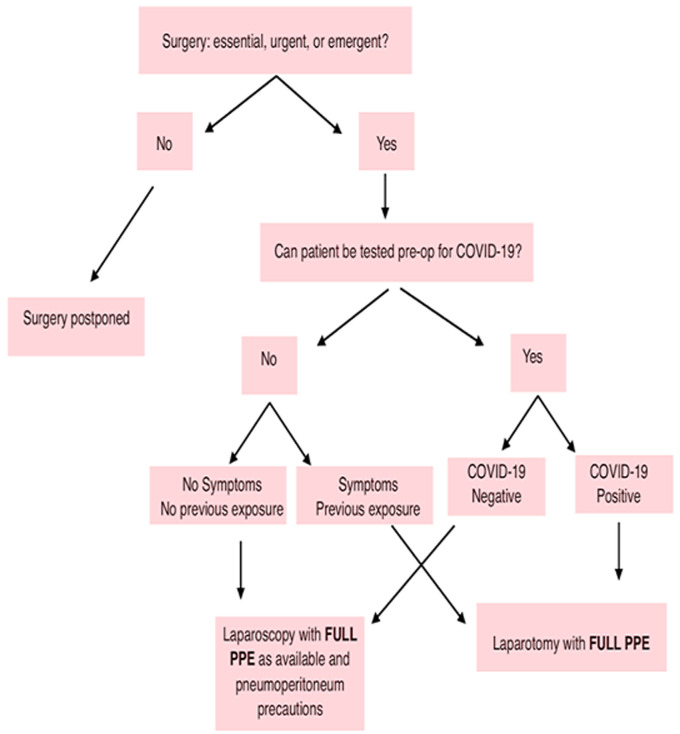
algorithm for surgical intervention of obstetrics and gynecologic cases

It is expected of gynecologists and obstetricians to review their present role by evaluating and mitigating risk to themselves, other staff and most importantly the patients. In addition, simulation training obstetrics emergencies will improve neonatal outcomes during shoulder dystocia; reduce time to surgical incision when required and improve fetomaternal outcomes when managing obstetrics hemorrhage. The recommendations for COVID-19 management in Africa by Ademuyiwa *et al*. (Table 2) as well as the algorithm in Figure 1 will equally contribute positively to obstetrics and gynecologic management during this period [57]. Putting into consideration several factors, the ongoing COVID-19 pandemic will significantly affect the obstetrics and gynecology practices. The routine essential services are disrupted due to limited or non-availability of PPEs and test kits. Moreover, fear of nosocomial infections by both healthcare providers and patients as well as possible redeployment of the healthcare workers across emergency health services pose additional challenges which would further have untold effects on our health system.

**Table 2:** recommendations for COVID-19 preparedness within the surgical, obstetric and anesthetic ecosystem in sub-Saharan Africa

Develop a clear plan for essential operations during the pandemic	Preserve hospital capacity to care for surgical and obstetric emergencies; postpone elective operations to preserve PPE, staff and facility capacity; adapting algorithms to categorize cases as elective, urgent or emergent and enforce them; trial of non-operative management of patients' conditions when appropriate; keep COVID+ patients geographically separate from other surgical patients; consider dedicating one operating room for COVID+ patient's use only if case burden is high; operating rooms used for COVID+ patients should be kept at neutral or negative pressure
Limit exposure of health care staff and prevent hospital transmission of SARS-CoV-19	Train staff on appropriate donning and doffing of PPE; encourage simulation and use of two providers for donning/doffing procedures; limit unnecessary patient and physician movement through the hospital; limit number of visitors; avoid involving students and trainees in patient care of COVID+ patients when possible; minimize the staff required in the hospital to preserve human resources; all staff including cleaners, laundry personnel and others should be provided with appropriate PPE; use surgical masks when caring for COVID-19 suspected or infected patients; launder all contaminated linens with detergent regularly; disinfect all hard surface areas regularly with 0.5% chlorine or 70% alcohol solution; enforce frequent and proper hand washing practices - alcohol-based hand rub is inexpensive and can be locally manufactured easily; develop care protocols and teams specifically for COVID response; consider establishing a COVID+ only operating room to be cleared of all materials; minimize aerosols during anesthesia: use regional anesthesia when possible, most senior provider should attempt intubation ensuring only absolutely essential personnel in OR during intubation; recover patients in OR; limit case duration, limit aerosolization during laparoscopy; consider use of COVID checklist for suspected/known COVID patients undergoing surgery; if reprocessing single use plastic materials, achieve high-level disinfection or sterilization
Conserve PPE and consumables	Develop a clear understanding of current stocks and supply chains; airborne precautions (N95 or PAPR) only required during aerosolizing procedures (intubation, bronchoscopy, NIPPV, high flow nasal cannula oxygen, nebulized medication administration); use droplet & contact precautions (surgical mask, eye protection, gown, gloves) for other patient encounters with suspected or known COVID patient; extended use of N95 masks is preferred to reuse of the same mask; N95 mask contamination may be reduced by covering with plastic face shield or surgical mask; do not decontaminate N95 respirators with chlorine or alcohol solution; if severe shortage, consider reprocessing N95 masks in 70°C oven for 30 minutes; wash reusable PPE (cloth hats, gowns, etc) between each use; cloth mask should be used as a last option only as it provides little protection against droplet or airborne particles
Plan to expand critical care and repurpose staff	Carefully consider if/how many ORs or PACUs could be repurposed for critical care needs; prepare providers to work outside their usual scope of practice; provide refresher trainings on ventilator management, critical care and COVID-specific care guidelines to providers who may be asked to work in different areas
Support staff wellness while assisting with difficult ethical considerations	Provide material and psychological resources to staff during this time of crisis; consider how needs such as HCW home isolation, child care, meal preparation or general stress management can be supported by hospital leadership; develop a plan in advance for managing resource shortages and determining scarce resource allocation; frontline healthcare workers should not have to make resource allocation decisions alone; provide compassion, empathy and respect for patients, family members and healthcare workers in this time of crisis

* ORs: operating rooms; PPE: personal protective equipment; PAPR: powered air-purifying respirator; PACU: postanesthesia care unit; NIPPV: nasal intermittent positive pressure ventilation; COVID: corona virus disease; SARS: severe acute respiratory syndrome; HCW: healthcare worker

### What is known about this topic

- The impact and implications of COVID-19 pandemic on the obstetrics and gynecology practice in Africa are yet to be fully explored;
- Obstetrical emergencies in patients who are suspected or confirmed COVID-19 cases are particularly complex.

### What this study adds

- This review has discussed extensively the potential implications of COVID-19 for obstetrics and gynecologic practice in Africa;
- The implications of the pandemic in several obstetrics and gynecology practices such as family planning services down to assisted reproductive techniques have been identified.
